# As a Novel Tumor Suppressor, LHPP Promotes Apoptosis by Inhibiting the PI3K/AKT Signaling Pathway in Oral Squamous Cell Carcinoma

**DOI:** 10.7150/ijbs.66841

**Published:** 2022-01-01

**Authors:** Shanshan Liu, Wenzhen Gao, Yupu Lu, Qin Zhou, Rongjian Su, Tomoka Hasegawa, Juan Du, Minqi Li

**Affiliations:** 1 Department of Bone Metabolism, School and Hospital of Stomatology, Cheeloo College of Medicine, Shandong University and Shandong Key Laboratory of Oral Tissue Regeneration and Shandong Engineering Laboratory for Dental Materials and Oral Tissue Regeneration, Jinan 250012, China.; 2 Department of Oral and Maxillofacial Surgery, Shandong Provincial Hospital Affiliated to Shandong First Medical University, Jinan 250021, China.; 3 Life Science Institute of Jinzhou Medical University, College of Basic Medicine of Jinzhou Medical University, Cell Biology and Genetic Department of Jinzhou Medical University, Key Lab of Molecular and Cellular Biology of the Education Department of Liaoning Province, Jinzhou 121001, China.; 4 Department of Developmental Biology of Hard Tissue, Graduate School of Dental Medicine, Hokkaido University, Sapporo, 060-8586, Japan.

**Keywords:** Oral squamous cell carcinoma, LHPP, proliferation, apoptosis, PI3K/AKT pathway

## Abstract

Oral squamous cell carcinoma (OSCC) refers to the malignant tumor of the head and neck with a highest morbidity. It exhibits a poor prognosis and unsatisfactory treatment partially attributed to delayed diagnosis. As indicated from existing reports, the protein histidine phosphatase LHPP acts as a vital factor in tumorigenesis in liver, lung, bladder, breast and pancreatic tumor tissues. Thus far, the functional mechanism of LHPP in OSCC remains unclear. DGE analysis, OSCC cell lines and OSCC cases were found that LHPP was down-regulated in OSCC tissues and cells compared with that in normal oral mucosa tissues and cells, and was closely related to OSCC differentiation. Cell counting Kit 8 test, EdU proliferation test, scratches test, invasion test, monoclonal formation test, mouse xenograft tumor model, HE staining and immunohistochemistry showed that LHPP inhibited OSCC growth, proliferation and migration *in vivo* and *in vitro*. GO and KEGG enrichment analysis, LHPP transcription factor analysis and flow cytometry found that LHPP promotes the apoptosis of OSCC by decreasing the transcriptional activity of p-PI3K and p-Akt. Finally, our results suggested that LHPP inhibited the progression of OSCC through the PI3K/AKT signaling pathway, indicating that LHPP may be a new target for the treatment of OSCC.

## Introduction

Oral squamous cell carcinoma (OSCC) refers to a highly frequent head and neck malignant tumor. It acts as a disease seriously jeopardizing human health. Each year, about 350,000 novel cases globally. OSCC takes up over 80% of oral and maxillofacial malignancies, and the incidence is on the rise 1-3. For its special tissue location, high degree of malignancy and strong infiltration, it tends to have regional lymph node metastasis. Besides, its prognosis is poor. Approximately 50% of oral carcinoma cases are at an advanced stage when they are diagnosed 4. Thus, the progression of this disease should be urgently clarified, and the diagnosis and treatment of OSCC should be optimized.

Phosphosarcosine phosphate histidine inorganic pyrophosphate phosphatase (LHPP), a novel tumor suppressor protein, was first confirmed as a tumor suppressor in 2018 5. LHPP is a highly conserved histidine phosphatase from worms to humans, originally found in swine brain tissues 6, 7. PHPT1, PGAM5 and LHPP are three known histidine phosphatases. The importance of the former two in tumor progression have been widely covered, but not LHPP. For the past few years, a lot of evidence has illustrated that LHPP is closely interrelated to the development of cancer. It was reported that the overexpression of LHPP in the mouse model of hepatocellular carcinoma prevented the decrease of liver function. Moreover, in patients with hepatocellular carcinoma, the low expression of LHPP is closely related with an increase in tumor malignancy and a decrease in overall survival 5. The latest research suggested that YTHDF2 could mediate the mRNA degradation of LHPP and NKX3-1 in a m6A-dependent manner for regulating the progression of prostate cancer tumors induced by AKT phosphorylation 8. A search in the databases of TCGA and the International Cancer Genome Consortium (ICGC) found 49 LHPP mutations, 49 LHPP mutations, (e.g., liver (1), skin (1), breast (1), bladder (1), stomach (2), head and neck (2), esophagus (2))^9^. The genome-wide association study (GWAS) also showed that the LHPP locus 10q26.13 (rs201982221, LHPP) is a significantly related locus in oral cancer carcinoma and pharyngeal cancer carcinoma. The current research on LHPP in carcinoma is limited to liver carcinoma, breast carcinoma, bladder carcinoma, papillary thyroid carcinoma and pancreatic carcinoma 10-13. But the role of LHPP in OSCC has not been studied.

Phosphatidylinositol-3 kinase (PI3K)/protein kinase B (PKB or AKT) signal transduction pathway refers to a pivotal signal transduction pathway in cells, displaying a close association with many kinds of malignant tumors occurrence and development (e.g., OSCC) 14, 15. According to existing studies, the phosphorylation level of AKT displays a close association with lymph node metastasis, prognosis and recurrence rate of oral carcinoma. The pro-apoptotic effects of some anti-cancer drugs are highly interrelated to the inactivation of the PI3K/AKT pathway 16, thereby demonstrating that inhibiting the PI3K/AKT signaling cascade may act as an effective tumor treatment strategy.

The present research aimed at clarifying the crucial role of LHPP in OSCC and exploring its underlying mechanism in depth. The results of *in vitro* and *in vivo* experiments here provide the first evidence for LHPP to play a tumor suppressor effect in OSCC, indicating that LHPP is a new diagnostic marker and therapeutic target for OSCC.

## Materials and Methods

### Data collection and preprocessing

According to GDC data portal (https://portal.gdc.cancer.gov/), the row count expression matrix and FPKM expression matrix of TCGA-HNSC cohort was acquired. Tumors in the oral cavity consist of, oral cavity, buccal mucosa, alveolar ridge, lip, hard palate and tongue. Cases with no clinical data or incomplete details were excluded. The STRING website (https://string-db.org/) was employed to discover their associations and establish a PPI network. Transcription factor prediction for LHPP was carried out based on GCBI database (https://www.gcbi.com.cn/gcanalyze/html/generadar/index).

### Bioinformatic analysis

We used the R package “edgR” for differentially expressed gene (DEG) analysis and the cutoff values for mRNAs exhibiting the differential expression were set as: |log2 FC|>1 and FDR<0.05. R package “ggplot2” was employed in terms of dot plots, boxplots and volcano-plots (R version 3.6.1).

### Tissue Sample

In total, 23 OSCC histopathological samples were obtained from School and Hospital of Stomatology, Cheeloo College of Medicine, Shandong University, patients provided informed consent before operation. The tumor grade was classified according to the WHO classification of histological differentiation. This research was carried out with the approval of the Ethics Committee of Shandong University School of Stomatology (No. 20210119). The researchers implemented the overall procedure according to the principles of the Declaration of Helsinki.

### Cell Culture and Reagents

The Shanghai Cell Bank of Chinese Academy of Sciences (Shanghai, China) offered oral squamous carcinoma cell lines (SCC15, SCC25). All OSCC cell lines were cultured in DMEM-F12 medium supplemented with 1% penicillin-streptomycin as well as 10% FBS (Gibco, Grand Island, NY, USA), and incubated in a 5% CO2, 37°C humidified incubator. Antibodies to LHPP, Akt, PI3K,p-Akt, p-PI3K, Bax, Bcl-2, Cleaved-Caspase 3 were purchased from Abcam (MA, USA). Proteintech (Wuhan, China) offered anti-GAPDH.

### Cell transduction and treatment

SCC15 and SCC25 cells were trans ducted with lentivirus: Ubi-MCS-3FLAG-SV40-puromycin (Genechem, Shanghai, China) at a multiplicity of infection (MOI) of 50 and 20 for 14h, HitransG P and HitransG A virus infection reagents were added, respectively, then co-culture with 1ug/ mL and 5ug/ mL puromycin for 14 days, finally, we obtained the SCC15 and SCC25 cells line that stably expressed LHPP. The full-length transcript of LHPP was in NM_022126.4. The OE-LHPP group is the cells that overexpress LHPP. The Vector group is the control blot for OE-LHPP, that is, it is infected with lentivirus but does not express LHPP. The OE-LHPP group cells were pretreated with 8μg/ml AKT activator SC79 or treated with 10 μM PI3K inhibitor LY294002, which were bought from MedChemExpress.

### Cell proliferation Assay

We seeded the NC, Vector, OE-LHPP group cells of SCC15 and SCC25 in the 96-well plate at the density of 5 × 10^3^ cells per well. After the incubation for 24 h, 48 h and 72 h, the viability of cells was ascertained based on the Cell Counting Kit-8 assays (CCK8, solarbio, Beijing, China).

The ethynyl deoxyuridine (EdU) incorporation assay was used to test the effect exerted by LHPP on the cell proliferation of SCC15 and SCC25. We seeded NC, Vector, OE-LHPP group cells of SCC15 and SCC25 in a 48-well culture plate at the density of 1× 10^4^ cells per well. Incubated for 24 hours, added EdU regents (RIBOBIO, Guangzhou, China) to each well and incubated for 2 hours. And then, fixed with 4% paraformaldehyde, cleaned by PBS, Apollo stained for 30 minutes, DNA stained for 30 minutes, and fully washed with PBS. Finally, photographed under an optical microscope (Olympus BX53, Tokyo, Japan) for cell proliferation analysis.

### Invasion Assay

We performed the transwell invasion assay for detecting the effect exerted by LHPP on the invasion ability of SCC15 and SCC25. First, the 8 µM pore-sized transwell chambers coated with matrigel were inserted in 24-well plate. Subsequently, 800 µL of a medium containing 10% FBS was introduced to the lower chambers. Next, cells suspended with serum-free DMEM-F12 were plated into the upper chamber. After the incubation was performed for different periods, the cells penetrating the cell membrane were fixed in 4% paraformaldehyde and received the 30 min staining with crystal violet. A cotton swab was used to take out the cells inside the upper chamber. Rinsed with PBS, then pictures were captured based on an optical microscope (Olympus BX53, Tokyo, Japan). Lastly, the cells were counted.

### Scratch Assay

The NC, Vector, OE-LHPP group cells of SCC15 and SCC25 were plated in 6-well plates, when cells reached 50% confluence, using a pipette tip to create a vertical scratch on the surface of the plate. Next, the mentioned plates were cleaned by using a serum-free medium. Afterwards, this study carried out the incubation of the plates by using DMEM medium with 1% FBS contained. Finally, at the specified time, we took pictures on the use of an optical microscope (Olympus BX53, Tokyo, Japan).

### Clone Formation Assay

The NC, Vector, OE-LHPP group cells of SCC15 and SCC25 were seed in 6-well plates 600 cells per well. Subsequently, the 14-day culture was performed, and the clone mass grew into a mass containing 50 cells. The cells were cleaned by using PBS. They received the fixing in 4% methanol and then the dying with 0.1% crystal violet. Furthermore, the colonies with over 50 cells covered were counted.

### Immunohistochemical (IHC) staining

The OSCC specimens and paraffin-embedded xenografts were made into 5 µm sections, fixed on the slides. The tissue on the slides were then dewaxed, hydrated, 1% BSA blocked for 20 minutes. LHPP antibody (1:200) was incubated overnight at 4 °C, and Goat-Anti-Rabbit antibody (1:300) was incubated at 37 °C for 1 h. Satisfied staining was obtained under the use of diaminobenzidine (DAB) (Sigma, Mo, USA). Lastly, we used methyl green to stain, gradient alcohol and xylene to dehydrate, a neutral adhesive to seal, and a fluorescence microscope (Olympus, Tokyo, Japan) to observe. The degree of LHPP expression in the tissue is shown in the image as the depth and area of the immunostain. Image analysis software (IPP 6.0) can be used to quantitatively measure the degree of IHC staining. As one of the commonly used indexes in quantitative analysis of IHC results, the mean optical density (MOD) value can better reflect the protein intensity expressed by positive cells. So, we used the MOD value calculated by IPP to reflect the LHPP expression.

### HE staining

After the samples were deparaffinized and hydrated, they were stained with hematoxylin for 10 min, cleaned with PBS. Then they were stained with eosin for 10 minutes and washed with PBS. Finally, dehydration and sealing with neutral gum, got the image under a fluorescent microscope.

### Immunofluorescence staining

First fix SCC15 and SCC25 cells with 4% formaldehyde, permeabilize with 0.5% Triton X-100, block with 5% BSA, LHPP antibody (1:200) was incubated overnight at 4 °C, and then incubation with the Goat-Anti-Rabbit antibody (1:300) for 1 h at room temperature, followed by incubated with DAPI for 10 min. Lastly, the fluorescent microscope (Olympus, Tokyo, Japan) was used for observing the cells.

### Apoptosis Flow-Cytometry Assay

The present study carried out the seeding process for cells in 6-well plates 3 × 10^5^ cells/ well, then the 24h culture. After the trypsinization, the cells were cleaned 2 times by using cold PBS and then received the suspension in 500 μl of binding buffer. The cells received the 20 min incubation in Annexin PI and V-FITC (KeyGEN, Nanjing, China) at ambient temperatures in the dark. Lastly, an investigation was conducted on the cells by flow cytometry with the use of Accuri C6 plus software (Becton Dickinson).

### Western blotting

RIPA lysis buffer (Beyotime, Beijing, China) with 1% protease and 1% phosphatase inhibitors contained was added to lysed cells and OSCC tissues. A BCA kit (Beyotime, Beijing, China) was employed to determine the protein concentration of the corresponding group. One-quarter volume of 5X SDS loading buffer was added to the respective sample, and then it received the 5 min heating at 100°C to denature the protein. The proteins (30μg per sample) were separated according to different molecular weights: the denatured proteins underwent 10-15% sodium dodecyl sulfate-polyacrylamide gel electrophoresis. Then, proteins of different molecular weights are transferred to the PVDF membrane. Blocking in 5% BSA at room temperatures for 1 hour, different primary antibody was introduced to the PVDF membrane and incubated at 4°C overnight. PVDF membrane were cleaned 3 times in 1% TBST and incubated with relevant secondary antibodies for 1 hour at room temperature. Washing in TBST, finally, captured by the FluorChem E System (ProteinSimple, Santa Clara, CA, USA).

### Real-time PCR analysis

Total RNA was acquired from cells with the use of Trizol (Invitrogen). We use a microspectrophotometer (LASPEC, Shanghai, China) to get RNA concentrations. And obtained the cDNA with the use of cDNA synthesis kit (Accurate Biology, Hunan, China). The SYBR green I Mix (Accurate Biology, Hunan, China) and real-time polymerase chain reaction (RT-PCR) detection system (Heal Force, Shanghai, China) to perform RT-PCR analysis. Lastly, GAPDH acted as an internal control indicator, and the data was analyzed with the use of the 2^-ΔΔCt^ method. The complete list of RT-PCR primer sequences was displayed in Table 1.

### Mouse xenograft tumor model

The Institutional Animal Care and Use Committee (IACUC) of Shandong University released the approval (No. 20210120) for the animal experiment. Athymic nude BALB/c female mice (Pengyue, Jinan, China) which were aged 4-6 weeks received the housing process within a specific pathogen-free environment based on free water and food as well as 12h light/12h dark cycle, and they were feed for 7 days to adapt to the environment. The mice received the random separation in 3 cohorts (n=6) and the subcutaneous injection in right upper limb back with 2×10^6^ SCC15 NC, Vector, OE-LHPP group cells /mice. Tumor size were detected once three days with the use of a slide caliper, the tumor volume was calculated with the use of 0.5×A×B^2^, where B represents the width and A denotes the tumor length. After feeded for 30 days, the mice were euthanized, the tumors received the isolation, weighed process, photographyed and immediate fixed by adopting 4% paraformaldehyde to conduct the following investigation.

### Statistical analysis

The data have the expression of the mean ± SD of 3 independent tests. Based on GraphPad Prism 6 software (San Diego, CA, USA), the statistical investigation was carried out. T-test was employed for testing the difference of the two groups. This study employed a one-way analysis of variance (ANOVA) for testing the distinctions of the groups.

## Results

### Low LHPP expression in OSCC tissues and cells

**Figure 1:**
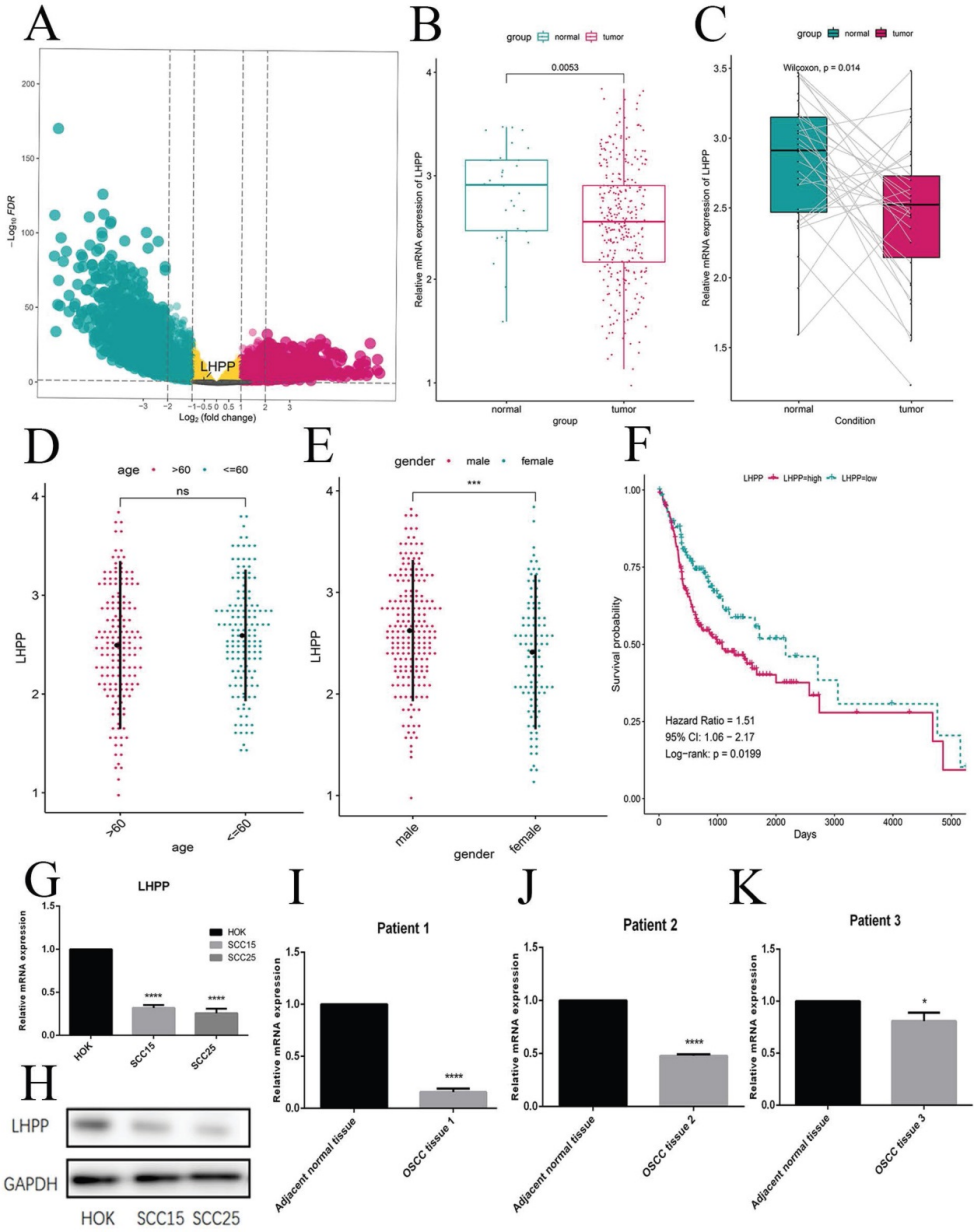
** Low LHPP expression in OSCC tissues and cells** (A) The Volcano plot indicated the genes exhibiting the differential expression for normal and OSCC sample. (B) In accordance with the boxplot, LHPP in the OSCC samples is lower than normal sample. (C) Pairwise boxplot suggested LHPP with a relatively low expressing level within tumor sample. (D, E) The expression status of LHPP between different age and gender, revealing the relationships of LHPP with age and gender. (F) The survival analysis indicated that LHPP is a risk factor and is related to the survival and prognosis of patient. (G, H) The mRNA and protein levels in normal oral keratinocytes HOK cells and OSCC cells was assessed by RT-PCR and Western blotting. (I, J, K) Western blot of the LHPP expression in OSCC tissues and adjacent normal tissues (n= 3). *P < 0.05, ***P < 0.001, ****P < 0.0001.

Following the criterion described before, this study included 335 OSCC samples from the TCGA-HNSC cohort, including 30 normal cases and 305 OSCC samples. According to the data preprocessing methods as previous study, a total of 16844 protein-coding genes were participated in the DEG analysis. To explore genes that are abnormally expressed in OSCC, we performed DEG analysis with the use of the criterion of |log2FC|>1 and FDR<0.05. The volcano plot presents the mRNAs exhibiting the differential expression (Figure 1A). From TCGA database and the DEG results, we found LHPP was down-regulated in OSCC. At the meanwhile, we observed the high expression of LHPP in normal cases but low expression in OSCC as well as paired cases (Figure 1B, C), thereby demonstrating that LHPP might adversely affect OSCC tumorigenesis and act as a tumor suppressor gene. These results also indicated that the differential expression of LHPP may be related to gender, but not age (Figure 1D, E). We performed a survival analysis of LHPP in the TCGA database, the log-rank value P=0.0199, P<0.05, it suggests that LHPP is associated with patient survival. Hazard Ratio=1.51, 95% CI:1.06-2.17, These results indicate that LHPP is a risk factor and is related to the survival and prognosis of patients (Figure 1F). And the mRNA and protein levels showed that normal oral keratinocytes HOK cells highly expressed of LHPP, while OSCC SS15 and SCC25 cells showed low expression (Figure 1G, H). We performed protein analyses of OSCC tissues and adjacent normal tissues from 3 cases to investigate the LHPP expression levels. As shown, the LHPP expression was lower in cancer tissues than that in normal oral mucosa tissue (Figure 1H, I, J). Given the mentioned results, it is speculated that LHPP is likely to play an anti-tumor role in OSCC.

### LHPP expression is closely related to OSCC differentiation degree

**Figure 2:**
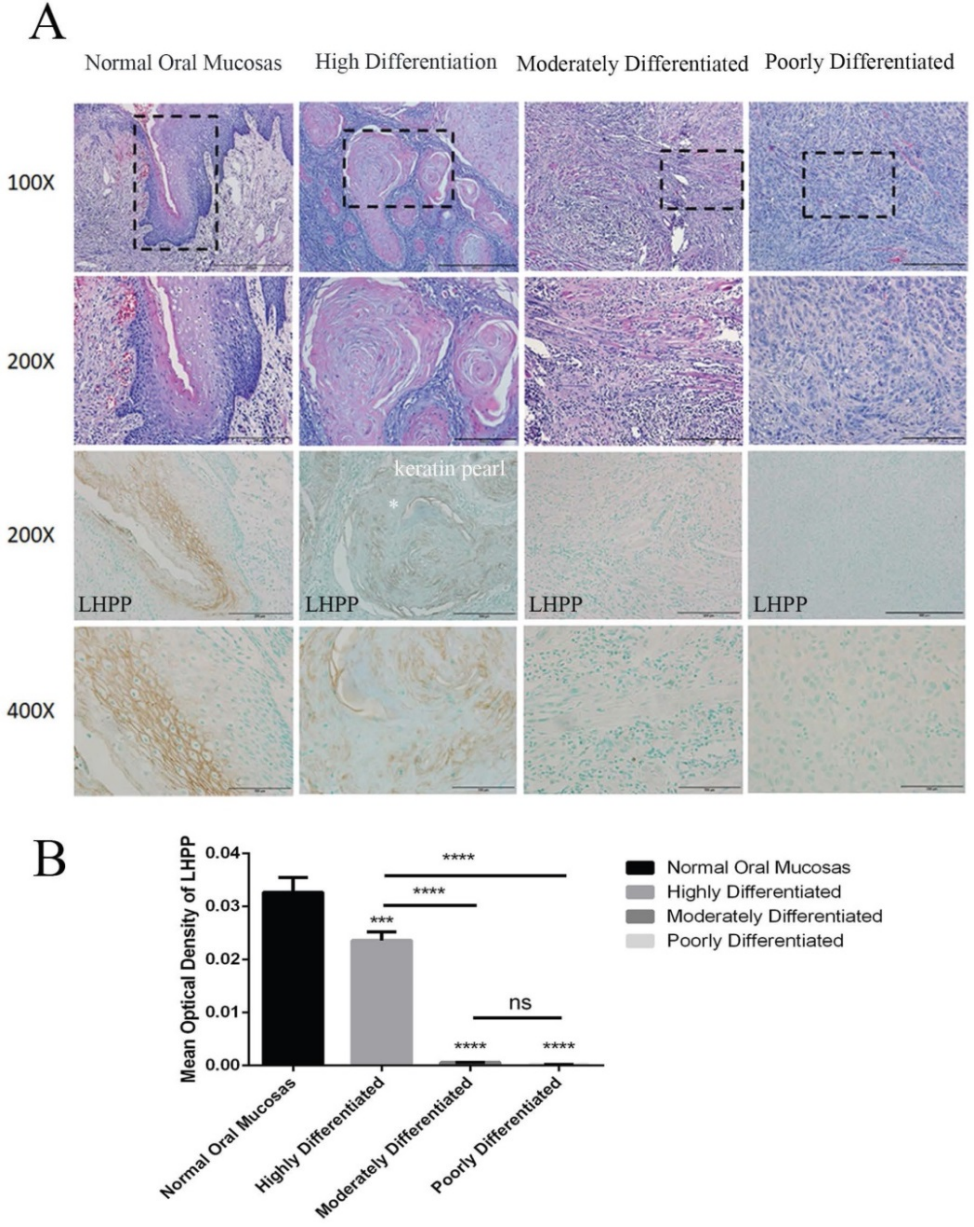
** LHPP expression is closely related to OSCC differentiation degree** (A) HE staining and IHC staining of LHPP in normal oral mucosas and differentiated OSCC tissues. (B) MOD analysis of the LHPP expression in normal oral mucosas and differentiated OSCC tissues. ***P < 0.001, ****P < 0.0001.

In contrast to normal tissues, LHPP expression was significantly reduced in OSCC, but there was also a small amount of LHPP expression in OSCC. We collected 23 OSCC tissues to analyze the relationship between LHPP expression and its differentiation degree. Microscopic observation showed that highly differentiated OSCC was similar to normal squamous epithelium, that is, there were different numbers of basal cells and squamous cells with keratin pearl appeared in the center of the carcinoma nest, and nuclear division was less; moderately differentiated squamous cell carcinoma had distinctive nuclear pleomorphism and nuclear division, infrequent keratosis; poorly differentiated squamous cell carcinoma was dominated by immature cells (Figure 2A). In Table 2, we further analyzed the expression of LHPP and the clinicopathological characteristics of OSCC. The results showed that the degree of differentiation of OSCC was negatively correlated with the expression (n=23, P=0.000002), whereas there were no differences in age, sex, tumor size, tumor location, and muscle invasion between groups (P > 0.05). IHC staining also illustrate that LHPP expression was significant in normal oral mucosas with an MOD of 0.033, decreased expression of LHPP in highly differentiated OSCC tissues with an MOD of 0.024, but almost no expression was found in moderately and poorly differentiated OSCC tissues with an MOD of 0.006 and 0.0018, indicating that LHPP can be used as a key factor to determine the degree of tumor differentiation (Figure 2A, B).The negative and positive controls used in immunohistochemistry were obtained from The Human Protein Atlas (http://www.proteinatlas.org/) 17. We could observe that LHPP was significantly expressed in normal liver tissues (Supplement 1A) and widely expressed in cytoplasmic and membrane, while LHPP was weakened in liver cancer tissues (Supplement 1B).

### Over-expressed LHPP cell lines were constructed

**Figure 3:**
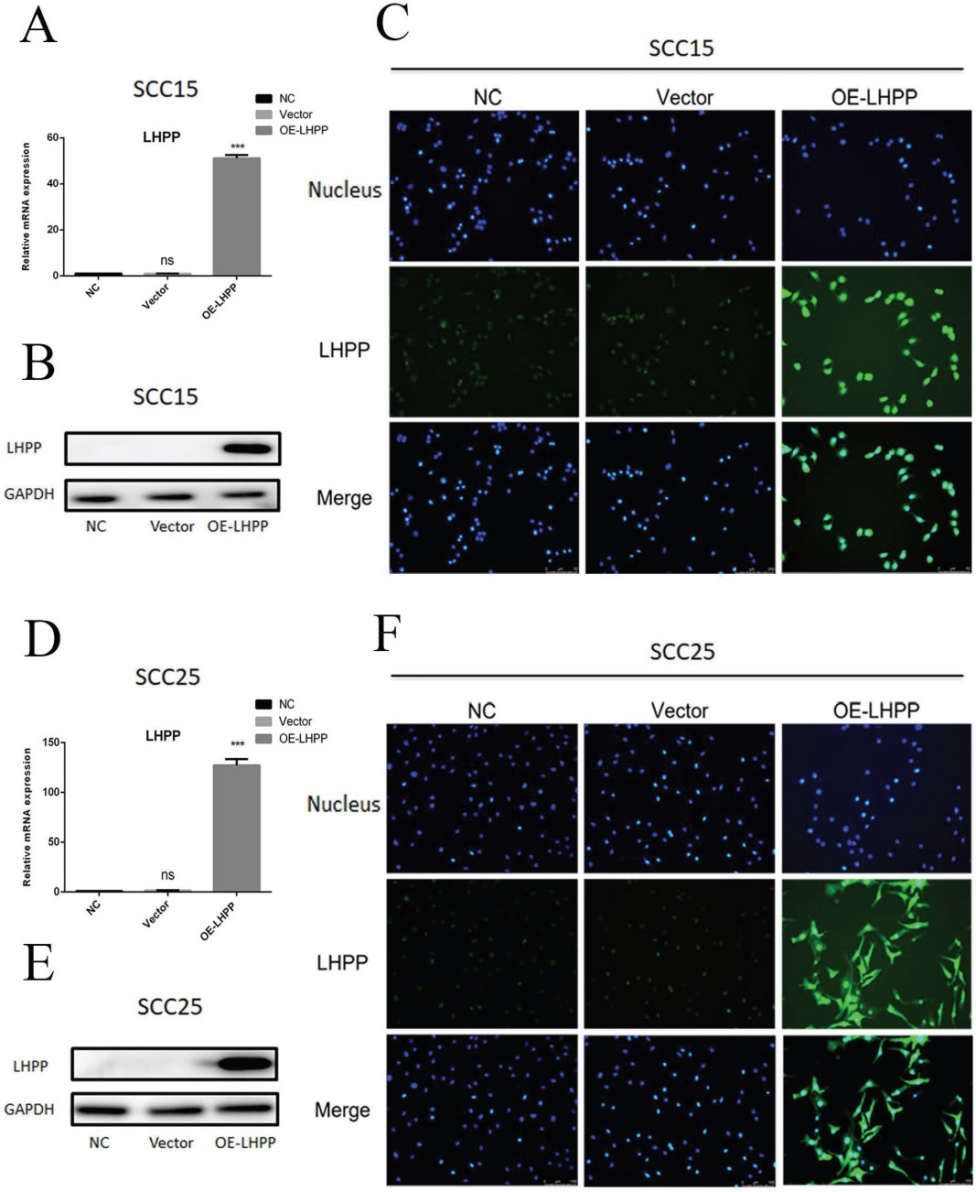
** Overexpressed LHPP cell lines were constructed** (A, B, D, E) RT-PCR and WB assay for the LHPP expression in OE-LHPP group of SCC15 and SCC25 cell lines. (C, F) IFC staining of LHPP in NC, Vector and OE-LHPP group in SCC15 and SCC25 cell lines (200X). ***P < 0.001.

Compared with the control group, transduction with LHPP lentivirus can significantly promote the expression of LHPP in both SCC15 and SCC25 cells at the level of mRNA and protein, the overexpression of LHPP has reached about 50-fold in SCC15 cells and about 125-fold in SCC25 cells (Figure 3A, B, D, E), and which was further verified by the results of immunofluorescence staining (Figure 3C, F). The high magnification immunofluorescence reaction images show that LHPP can not only express in cytoplasmic, but also in nuclear (Supplement 2A, B). It's possibly due to the lentiviral vector integrating the LHPP gene into the host chromosome, and the nucleus is small, so there is relatively strong fluorescence in the nucleus of the OE-LHPP group. In addition, according to the prediction of The Human Protein Atlas (https://www.proteinatlas.org/), the main location of LHPP protein in the cell are cytoplasm and nucleus.

### Over-expression of LHPP inhibited cell proliferation, migration and invasion in OSCC cells

**Figure 4:**
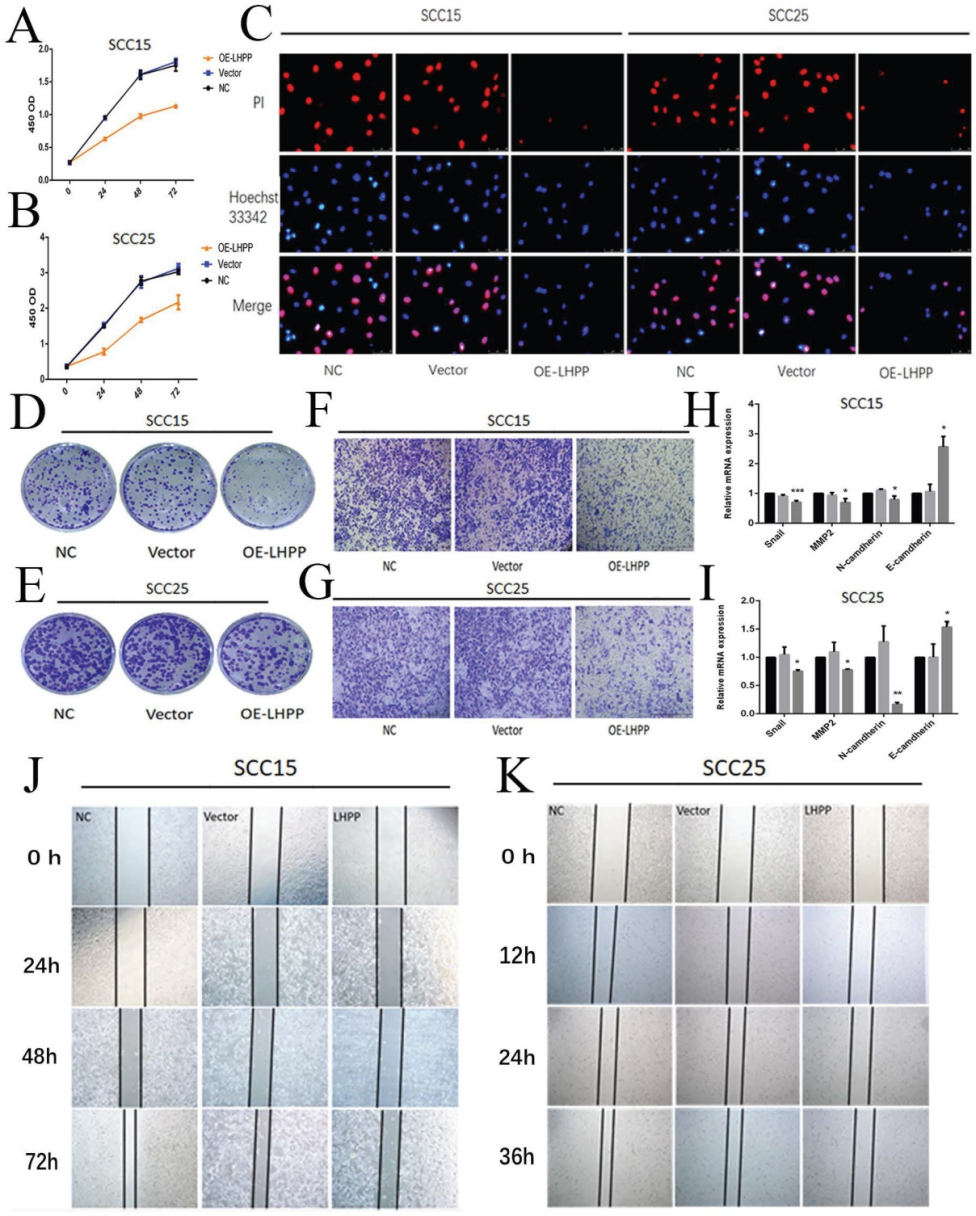
** Over-expression of LHPP inhibited cell proliferation, migration and invasion in OSCC cells** (A, B) Cell viability of SCC15 and SCC25 in NC, Vector, OE-LHPP groups cells for 24, 48 and 72 hours. (C) EdU assay of cell proliferation of SCC15 and SCC25 cells over-expressed LHPP or not for 24h. (D, E) The colony formation assay of SCC15 and SCC25 in NC, Vector, OE-LHPP groups for 14 days. (F, G) Transwell analysis for invasion of SCC15 for 24 h and SCC25 cells for 12 h. (H, I) RT-PCR analysis of snail, MMP2, N-cadherin and E-cadherin in the NC, Vector and OE-LHPP groups of SCC15 and SCC25. (J, K) The wound healing detection of SCC15 for 24, 48, and 72 hours and SCC25 cells for 12, 24, and 36 hours. *P < 0.05, **P < 0.01, ***P < 0.001.

Next, we explored the effect of LHPP on cell function. First, cell viability was assessed using CCK-8, it was found that the upregulation of LHPP led to decrease cell viability (Figure 4A, B). Then, EdU cell proliferation experiments and colony formation assays showed that proliferation of SCC15 and SCC25 was inhibited in the over-expressed LHPP group than control and vector group (Figure 4C, D, E). The number of cells that passed through the membranes were counted using IPP6.0 software (Supplement 2C, E). Furthermore, the OE-LHPP group showed significantly inhibited migration and invasion by scratch test and transwell test (Figure 4F, G, J, K). The statistical analysis of the relative wound closure in SCC15 and SCC25 cells was shown in Supplement 2D, F. Finally, we performed RT-PCR to analysis the expression of snail, MMP2, N-cadherin and E-cadherin in mRNA level. The results showed that compared with control and vector group, OE-LHPP group can decrease the expression of snail, MMP2 and N-cadherin, whereas the expression of E-cadherin showed the opposite result (Figure 4H, I).

### Overexpressed LHPP restricted the growth of xenograft tumors *in vivo*

**Figure 5:**
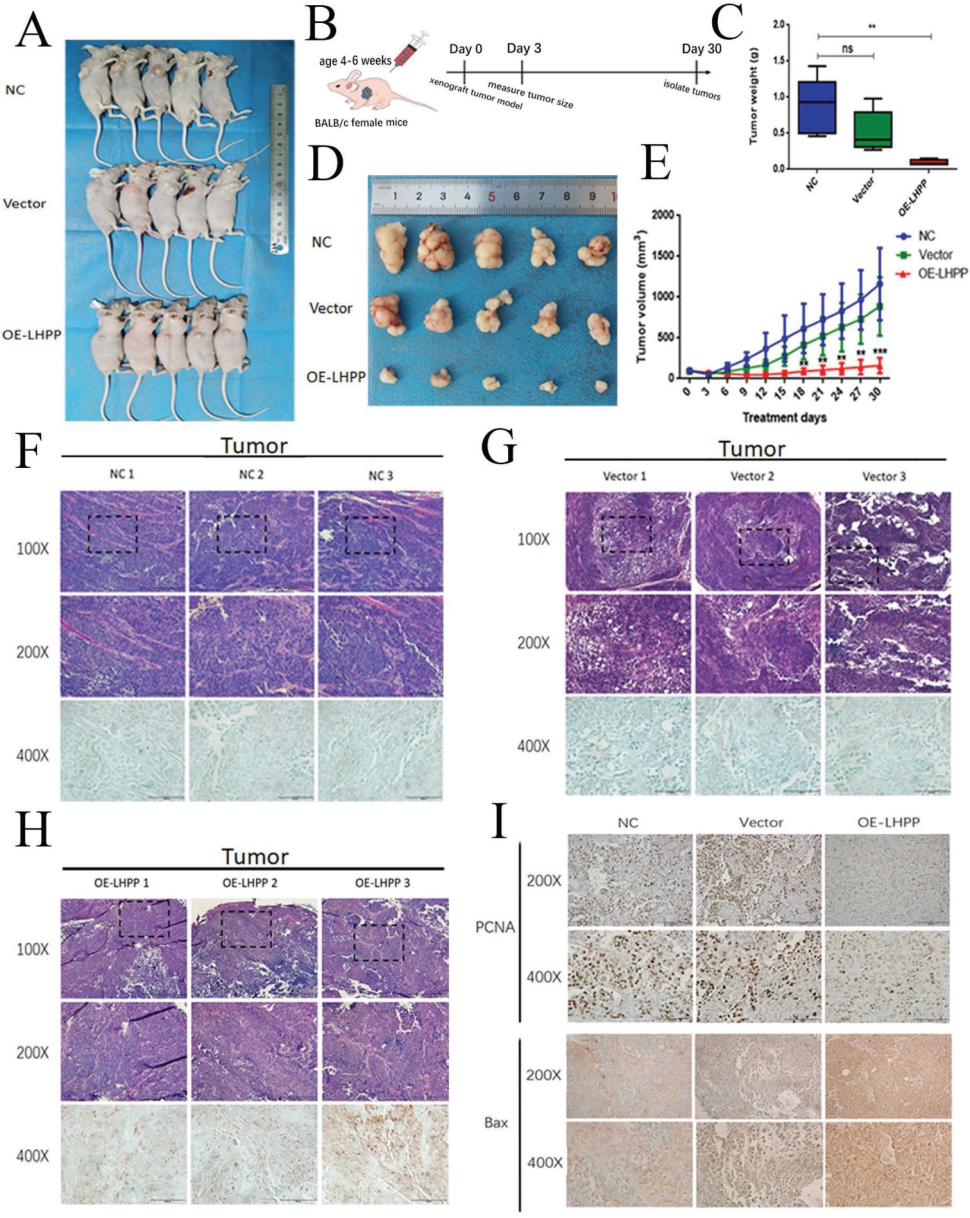
** Overexpressed LHPP restricted the growth of xenograft tumors *in vivo*** (A) Images of the xenograft tumors from BALB/c mice induced with NC, Vector, OE-LHPP groups cells. (B) Technical roadmap of tumor formation experimental procedures in nude mice. (C) Changes in tumor weight within 30 days after injection of cells in BALB/c mice. (D) The tumors peeled from BALB/c mice are presented. (E) Tumors volume after being inoculated with different groups of SCC15 cells for 30 days. (F, G, H) Representative images of HE and LHPP IHC staining of xenografts in NC, Vector and OE-LHPP groups. (I) Representative images of PCNA IHC staining of xenografts in NC, Vector and OE-LHPP groups. **p<0.01, ***P < 0.001.

According to the results of CCK8, EdU, clone formation and transwell experiment results, LHPP has a more prominent inhibitory effect on proliferation in SCC15 cell lines, so we used the nude mouse implanted subcutaneously with NC, Vector and OE-LHPP groups of SCC15 to study the anticancer effect of LHPP *in vivo*. The average tumor volume and weight of the OE-LHPP group were significantly smaller than that of the control group and the vector group (Figure 5A, C, D, E). Furthermore, HE and IHC staining showed that LHPP expression most in OE-LHPP group which was transfected with lentivirus (Figure 5F, G, H). Representative images of PCNA IHC staining of xenografts in NC, Vector and OE-LHPP groups also show that overexpression of LHPP can significantly reduce PCNA expression (Figure 5I). MOD analysis of the PCNA expression was shown in Supplement 2G. Therefore, these results indicate that overexpression LHPP limits the growth of xenograft OSCC cells *in vivo*.

### Over-expression LHPP promoted cells apoptosis by suppressing PI3K/AKT signaling pathway

**Figure 6:**
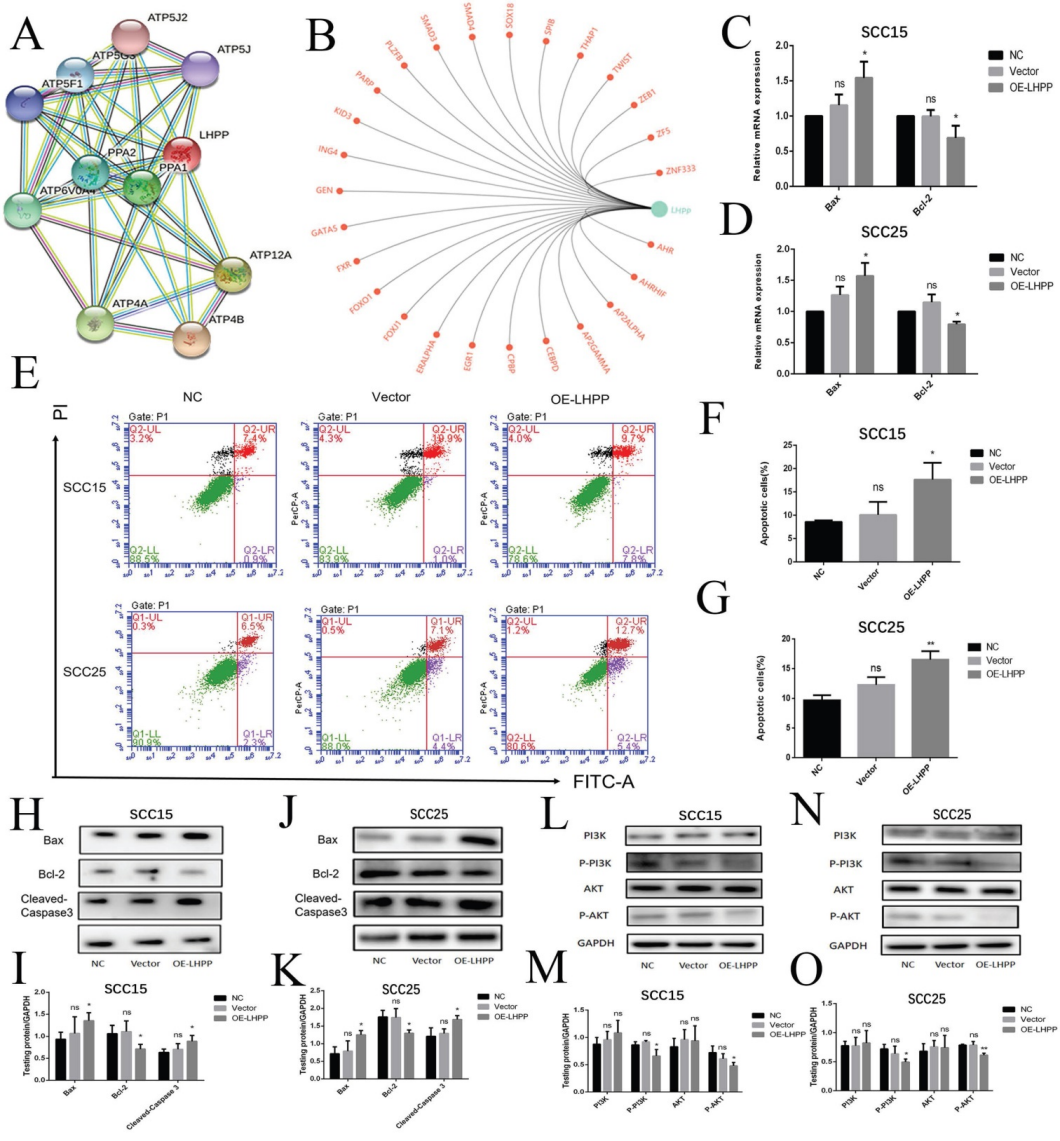
** Over-expression LHPP promoted cells apoptosis by suppressing PI3K/AKT signaling pathway** (A) The network revealed the regulating relationship among LHPP, ATP4B and other related genes. (B) LHPP transcription factor prediction for LHPP by GCBI website. (C, D) RT-PCR analysis of the bcl-2 and bax expression level in NC, Vector and OE-LHPP group of SCC15 and SCC25 cell lines. (E, F, G) Flow cytometry to analyze apoptosis of OSCC cell. The apoptosis of OE-LHPP group was more significant than that of NC group and Vector groups. (H, I, J, K) WB assay for the bax, bcl-2 and cleaved-Caspase 3 expression in SCC15 and SCC25 cell lines. (L, M, N, O) The protein expression of AKT, p-AKT, PI3K, p-PI3K were estimated by performing the WB assay. *p<0.1, **p<0.01, ***P < 0.001.

To further predict the role of LHPP and its related genes in depth, we performed GO and KEGG enrichment analysis in STRING, it was suggested that LHPP protein mainly interact with ATP synthase α/β family and phosphorylase super family (Figure 6A), involved in regulating oxidative phosphorylation and energy metabolism and signaling pathways. Furthermore mitochondrial oxidative phosphorylation in cellular energy production, production of active oxygen substances, and starts to critically impact cell apoptosis 18. LHPP transcription factor analysis for LHPP showed that LHPP was closely related to the transcription of PARP and FOXO1 (Figure 6B), which are important transcription factors in the process of apoptosis. We also explore the correlation between the expression of LHPP and apoptotic proteins in the TCGA dataset, it is shown that: pro-apoptotic protein bax (P<0.01), bid (P<0.001) and caspase-9 (P<0.01) mRNA expression are positively correlated with the expression of LHPP (Supplement 3A, B). Therefore, we will focus on exploring the relationship between LHPP and apoptosis of OSCC cells. As shown (Figure 6C), the apoptosis rate increased in the SCC15 and SCC25 OE-LHPP group cells. In SCC15 cells, the early apoptotic rate in the NC group was 0.9%, and the late apoptotic rate was 7.4%; the early apoptotic rate in the Vector group was 1%, and the late apoptotic rate was 10.9%; the early apoptotic rate in the OE-LHPP group was 7.8%, and the late apoptotic rate was 9.7%. In SCC25 cells, the early apoptotic rate in the NC group was 2.3%, and the late apoptotic rate was 6.5%; the early apoptotic rate in the Vector group was 4.4%, and the late apoptotic rate was 7.1%; the early apoptotic rate in the OE-LHPP group was 5.4%, and the late apoptotic rate was 12.7% (Figure 6E, F, G). Cleaved-Caspase 3, bax and BCL-2 serve as important indicators during the process of apoptosis. The mRNA expression shown that LHPP overexpression elevated the level of bax and bcl-2 mRNA level was opposite (Figure 6C, D). Meanwhile, the increased protein expression of bax and cleaved-caspase 3 and lower bcl-2 protein expression in the OE-LHPP group suggested that LHPP induced OSCC cells apoptosis (Figure 6H, I, J, K). Representative images of bax IHC staining of xenografts in NC, Vector and OE-LHPP groups also show that overexpression of LHPP can significantly induce the expression of pro-apoptotic protein bax (Figure 5I). MOD analysis of the bax expression was shown in Supplement 2H. Finally, the less expression level of p-AKT and p-PI3K in OE-LHPP group indicated that over-expression LHPP elevated cells apoptosis rate via suppressing PI3K/AKT signaling pathway.

### PI3K/ AKT play a great role in the promotion mechanism of LHPP on oral squamous carcinoma cells apoptosis

**Figure 7:**
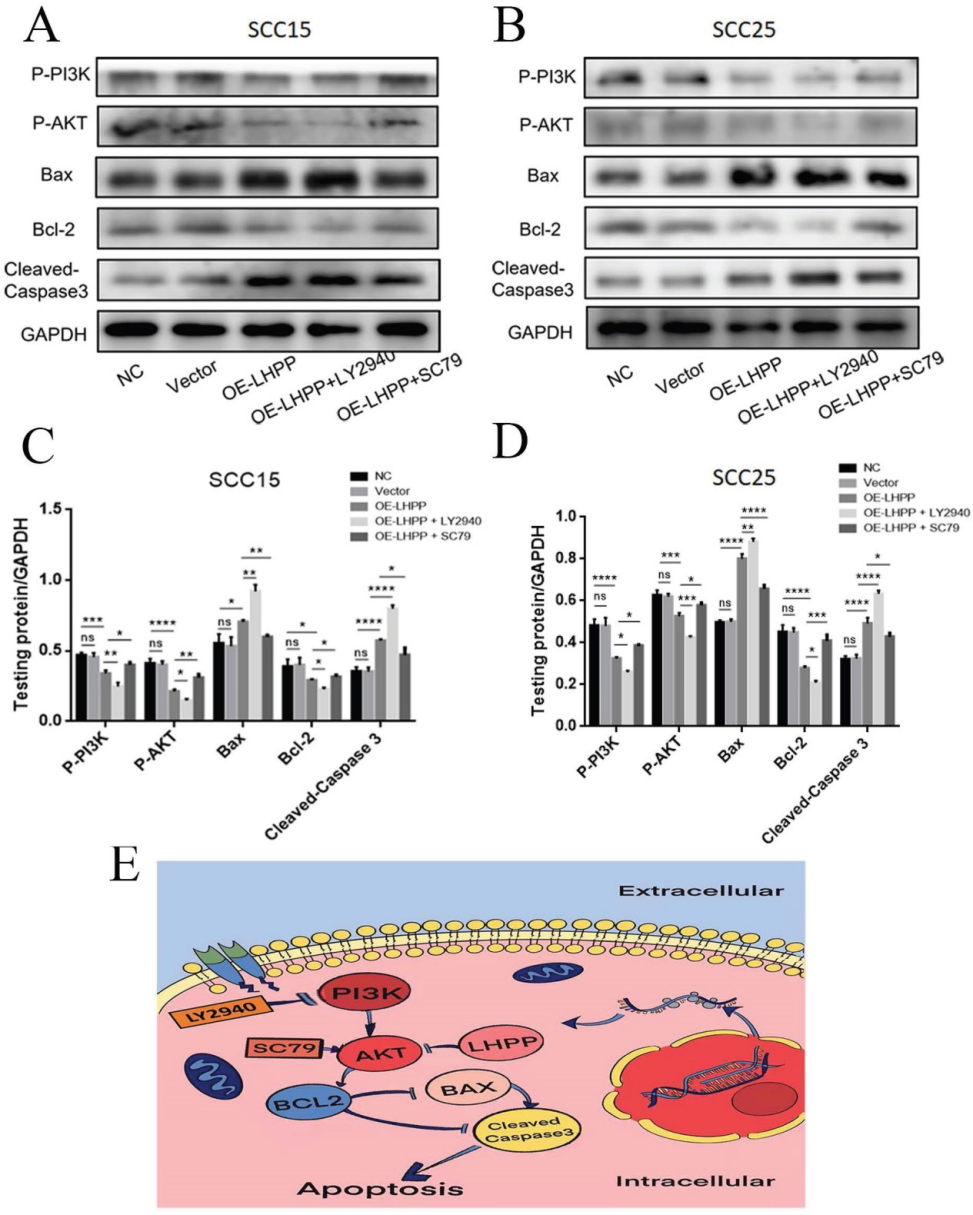
** PI3K/ AKT play a great role in the promotion mechanism of LHPP on oral squamous carcinoma cells apoptosis** (A, B) The protein expressions of bax, bcl-2, cleaved-caspase 3, PI3K, p-PI3K, AKT and p-AKT were analyzed by performing the Western blotting assay. (C, D) The statistical investigation of the protein expression in SCC15 and SCC25 cells. (E) A predicted cellular signaling pathway model showing LHPP as tumor suppressor. *p<0.1, **p<0.01.

To define the signaling mechanism triggered by LHPP affected the apoptosis of OSCC cells pathway. The AKT activator pretreatment in the OE-LHPP group noticeably reduced the levels of bax and cleaved-caspase 3 and elevated the level of bcl-2, p-AKT and p-PI3K in contrast to OE-LHPP group in cancer cells, while the inhibitor of PI3K treatment in OE-LHPP group obvious increased the expressions of bax and cleaved-caspase 3 and reduced the expression of bcl-2, p-AKT and p-PI3K compared with OE-LHPP group in SCC15 and SCC25 cells (Figure 7A, B, C, D). In general, after treatment with AKT activator, the promotion effect of overexpression of LHPP on cell apoptosis was invalid, in contrast, after treatment with PI3K inhibitor, the promotion effect of overexpression of LHPP on cell apoptosis was more obviously. According to the mentioned results, LHPP could increase the apoptosis rate of cells via PI3K/AKT pathway, we pretreated the OE-LHPP group with SC-79 (an activator of AKT) in 8µg/ml and treated with LY294002 (an inhibitor of PI3K) in 10 μM.

## Discussion

OSCC as one of the most common malignancies in the world, known for its high mortality and short survival time. The incidence of systemic malignant tumors accounts for about 2%-4%, and about 80% of malignant tumors occur in the oral and maxillofacial region, and the incidence has an increasing trend 1-3. Meanwhile, its pathogenesis is complicated 19. Therefore, to discover the pivotal factors in the occurrence and development of OSCC and clarify its mechanism of function is conducive to the discovery of potential markers and therapeutic targets for OSCC diagnosis. In our study, we wanted to find out the key factors affecting the progression of OSCC and explore its biological function and clinical significance. Difference analysis showed abnormal expression of mRNA, including down-regulated expression of LHPP in cancer. Analysis of clinical tissue samples illustrated that compared with adjacent healthy tissues the LHPP expression level was lower in tumor tissues, and the expression status of LHPP was closely related to the differentiation degree of OSCC. Next, according to its bioinformatics analysis, it was predicted that LHPP might be involved in the regulation of OSCC apoptosis. Then experimental verifications including CCK8 assay, EdU cell proliferation assay, clone formation assay, cell invasion and migration assay, RT-PCR, western blot, and flow cytometry cell apoptosis assay *in vitro* and xenograft tumors *in vivo* were conducted. Lastly, we discovered that as a tumor suppressor, LHPP can not only reduce cell viability, inhibit cell proliferation, migration and invasion, but also participate in cell apoptosis via PI3K/ Akt pathway.

LHPP is a histidine phosphatase that dephosphorylates proteins containing histidine phosphate, besides, its action is opposite to histidine kinase. Two mammalian histidine-kinase enzymes (NME1 and NME2), three histidine-kinase phosphatases (PHPT1 PGAM5, and LHPP) and several substrates have been reported. Their interaction is closely interrelated to the rapid proliferation, invasion and metastasis to adjacent tissues, and poor prognosis of tumors 20. Additionally, histidine phosphate plays a broad range of roles in protein and cell functions, including regulating cell growth, cycle, phagocytosis and ion channel activity 21-23.

At present, LHPP has been rarely studied, and LHPP has been scarcely evidenced to act as phosphatase in the development of carcinoma. Previous researches have found that LHPP is associated with chronic oxidative stress and mitochondrial dysfunction. Whole genome sequencing showed that LHPP gene was a risk factor for alcohol dependence and severe depressive disorder 24, 25. Several GWAS revealed LHPP as a susceptibility gene for primary open-angle glaucoma, oral carcinoma, pharyngeal carcinoma, and acute lymphoblastic leukemia 26-28 .The researchers found that LHPP mutation, decreased expression, and elevated levels of histidine phosphorylation were the key factors for tumor genesis in esophageal, skin, head and neck, stomach, breast, bladder, lung, liver, and pancreas tumor tissues 29.Therefore, LHPP is expected to be one of the effective markers and potential therapeutic targets in the diagnosis of OSCC cancer, and its function and mechanism of function remain to be further studied. In this study, according to the TCGA database and DEG analysis, LHPP is down-regulated in OSCC and is highly expressed in normal samples, and the trend in paired samples is the same, the survival analysis indicated that LHPP is a risk factor and is interrelated with the survival and prognosis of patients. Moreover, DEG analysis also indicated a very interesting observation that the differential expression of LHPP may be related to gender, it is shown that LHPP expression was significantly upregulated in males compared to females. But the relationship between LHPP expression and gender in 23 OSCC patients showed no statistical significance (Table 2), which may be due to the small amount of clinical data we have. Collectively, the mRNA and protein levels shown that epithelial cells of normal oral mucosa highly expressed of LHPP and OSCC cells was less expressed. We also performed the expression level of LHPP protein of OSCC tissues and adjacent normal tissues from 3 cases. As shown, compared with normal oral mucosa tissue the LHPP expression was lower in cancer tissues, IHC staining also indicated LHPP expression is closely related to OSCC differentiation, suggesting that LHPP might play a negative role in OSCC tumorigenesis and function as a tumor suppressor gene.

According to existing researches, LHPP is capable of inhibiting cervical carcinoma cells from the proliferation and metastasis, as well as facilitating their apoptosis by AKT regulation 30. Complying with this finding in bladder carcinoma, LHPP down-regulation can enhance cell viability and colony formation through AKT/p65 pathway 31. However, few studies have reported the role of LHPP in human OSCC, and its underlying mechanism remains unclear. According to this research, LHPP overexpression could inhibit OSCC cells from growth and metastasis. Besides, we reported the negative relationship between LHPP expressing levels and apoptosis rate in OSCC cells. Since the bax, bcl-2, cleaved-caspase 3, PI3K, p-PI3K, AKT, p-AKT act as the vital factors in the pathway, the expressions of the mentioned oncogenes in the OE-LHPP group cells were ascertained. Likewise, according to the data of this study, LHPP overexpression elevated the expressions of bax and cleaved-Caspase 3 and reduced the expressions of bcl-2, p-PI3K and p-AKT. Furthermore, after pretreatment with AKT activator SC79, the promotion effect of overexpression of LHPP on cell apoptosis was invalid, and in contrast, after treatment with PI3K inhibitor LY294002, the promotion effect on cell apoptosis was more obviously. In recent years, many advances have been made in the role of PI3K/AKT inhibitors in OSCC. For example, PI3K inhibitors could promote the apoptotic activity of the anticancer drugs cisplatin, 5-fluorouracil or docetaxel in OSCC cell lines 32, 33. PI3K inhibitors such as PI-103, PI-828 and PX-866 may be developed as potential therapeutic agents for effective treatment of OSCC patients 34. Dox or AD198 combined with PI3K/AKT pathway inhibitors are more effective in treating OSCC 35. An increasing number of PI3K/Akt inhibitors have anticancer effects in preclinical studies 36, 37.A decade ago, more than 30-40 compounds targeting the PI3K pathway were in clinical trials, and in recent years, most have received FDA approval. Such as the PI3K inhibitors BYL719 (alpelisib), CAL101 (idelalisib) and BAY 80-6946 (copanlisib), and the mTOR inhibitors RAD001 (everolimus) and CCI-779 (temsirolimus), have been approved for the treatment of various malignancies 38. The results from the present study suggest that LHPP is likely to act as a novel diagnostic and therapeutic target in terms of OSCC, and complementary use of PI3K inhibitors may further increase the antitumor effect.

This study still has some limitations: the validation cohort is very limited is due to the fact that as a specialized hospital, our hospital provides patients with authoritative diagnosis, but the patients choose to go to a general hospital for surgery, lacking follow-up information in the later period. And due to the lack of research on LHPP at the present stage, its specific mechanism is still unclear, so the specific role of LHPP in the AKT pathway has not been further studied, this study is relatively superficial. In future experiments, we will further explore the mechanism of upstream regulation of LHPP.

## Conclusions

In this context, we mainly elucidate LHPP as a new tumor suppressor, which can not only restrict cell proliferation, migration and invasion but also accelerate cell apoptosis via suppressing PI3K/AKT pathway in OSCC. And the expression of LHPP displays a close association with the differentiation of OSCC, thereby demonstrating that LHPP can act as a target to diagnose and treat OSCC.

## Supplementary Material

Supplementary figures.

## Figures and Tables

**Table 1:** Primer sequences for RT-PCR.

Resource	Gene name	Primer sequence: (5--3)
Human	GAPDH	Forward: CCTGCACCACCAACTGCTTA
		Reverse: GGCCATCCACAGTCTTCTGAG
Human	LHPP	Forward: CAAACTGTGTGGTAATTGCAGA
		Reverse: CCAGAGGTCTCCTTGTAGTAAC
Human	Snail	Forward: CCTTCGTCCTTCTCCTCTACTT
		Reverse: GCTTCGGATGTGCATCTTGA
Human	MMP-2	Forward: TGCTGGAGACAAATTCTGGA
		Reverse: TTGGTTCTCCAGCTTCAGGT
Human	N-cadherin	Forward: CGATAAGGATCAACCCCATACA
		Reverse: TTCAAAGTCGATTGGTTTGACC
Human	E-cadherin	Forward: AGTCACTGACACCAACGATAAT
		Reverse: ATCGTTGTTCACTGGATTTGTG
Human	Bax	Forward: CGAACTGGACAGTAACATGGAG
		Reverse: CATCTGGTCTGGAGTACGTATC
Human	Bcl-2	Forward: AAGAATGGCCAGACAATGAATG
		Reverse: CATCTGGTCTGGAGTACGTATC

**Table 2:** The relationship between LHPP expression and clinicopathological characteristics in patients with OSCC (23 cases).

Characteristics	LHPP gene expression (no. of patients)		Total	χ2	P-value
	Low	High			
No.	13	10	23		
Age (years)				1.051	0.305
≤60	5	6	11		
>60	8	4	12		
Gender				2.253	0.133
Male	5	7	12		
Female	8	3	11		
Longest tumor dimension (cm)				0.306	0.58
≤1.5	8	5	13		
>1.5	5	5	10		
Tumor location				0.212	0.645
Tongue	9	6	15		
Buccal/ Palate	4	4	8		
Differentiation				23	**0.000002**
Well-moderate	0	10	10		
Moderate/Poor	13	0	13		
Muscular invasion				1.704	0.192
Yes	6	2	8		
No	7	8	15		

Bold font shows that LHPP expression is significantly correlated with clinicopathological characteristics.

## Abbreviations

OSCC: Oral squamous cell carcinoma
LHPP: Phosphosarcosine phosphate histidine inorganic pyrophosphate phosphatase
ICGC: International Cancer Genome Consortium
GWAS: Genome-wide Association Study
PI3K: Phosphatidylinositol-3 kinase
IACUC: Institutional Animal Care and Use Committee
DEG: Differentially expressed gene
