# Supplementary material for: As a Novel Tumor Suppressor, LHPP Promotes Apoptosis by Inhibiting the PI3K/AKT Signaling Pathway in Oral Squamous Cell Carcinoma
Source: Int J Biol Sci. 2022 Jan 1;18(2):491–506. doi: 10.7150/ijbs.66841 (PMC8741864; doi:10.7150/ijbs.66841)
Supplement: Supplementary file 1 — Supplementary figures. [file ijbsv18p0491s1.pdf]

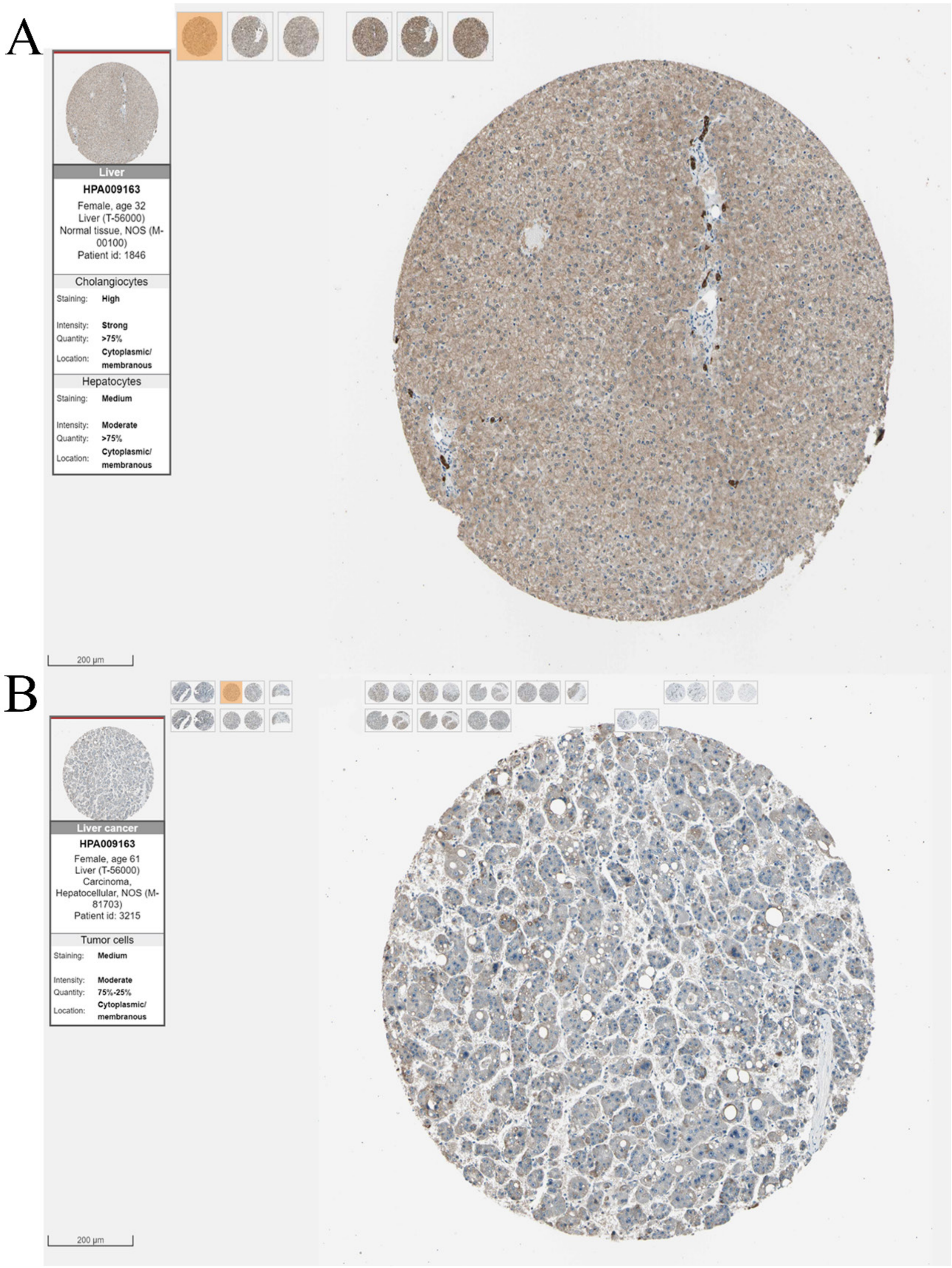

Supplement 1. (A, B) Representative images of LHPP IHC staining of normal liver tissues and liver cancer tissues from The Human Protein Atlas (http://www.proteinatlas.org/).

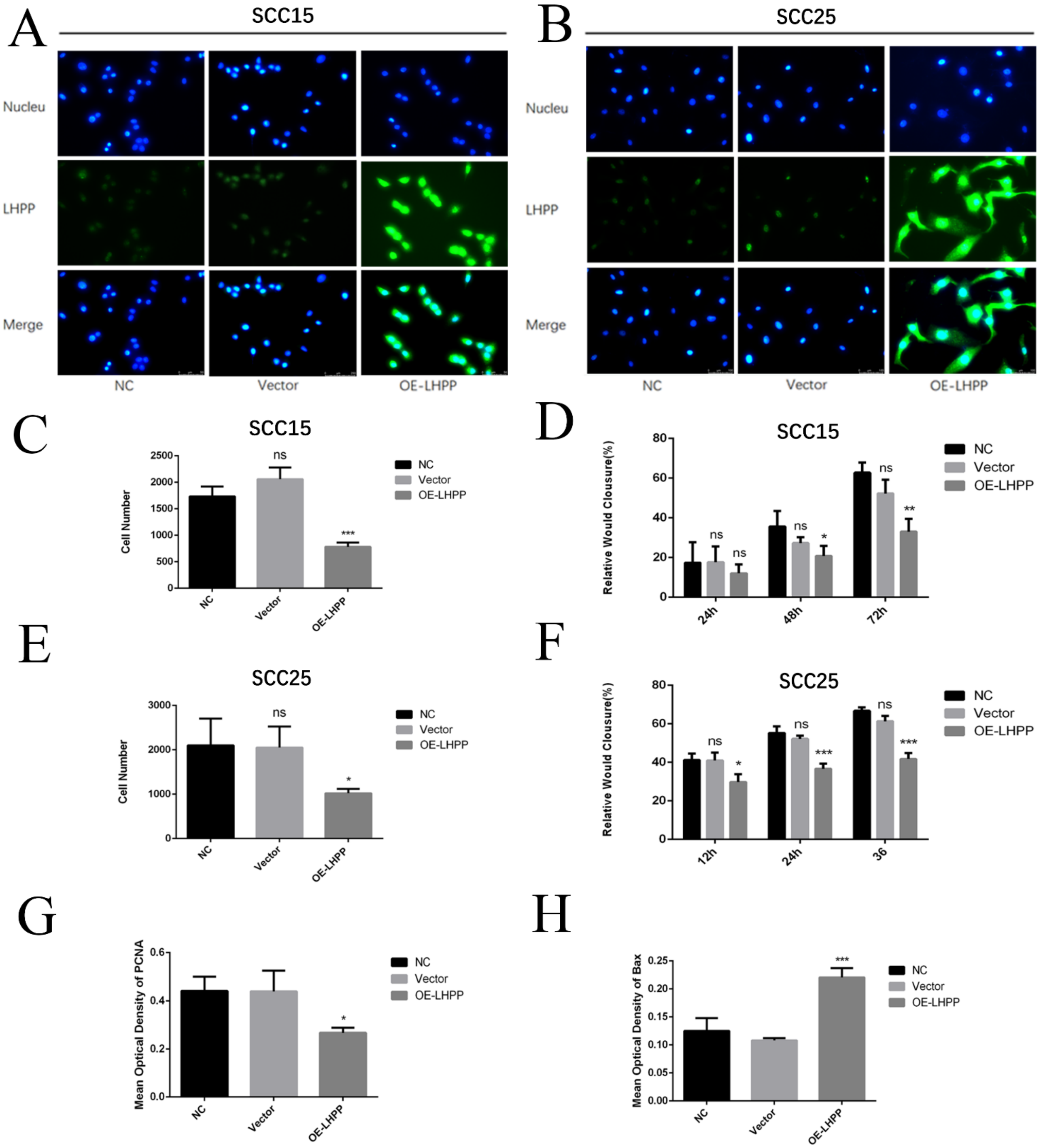

Supplement 2. (A, B) IFC staining of LHPP in NC, Vector and OE-LHPP group in SCC15 and SCC25 cell lines (400X). (C, E) The number of cells that passed through the membranes were counted using IPP6.0 software. (D, F) The statistical analysis of the relative wound closure in SCC15 and SCC25 cells. (G, H) MOD analysis of the PCNA and Bax expression in xenograft tumors. *p<0.1, **p<0.01, ***P < 0.001.

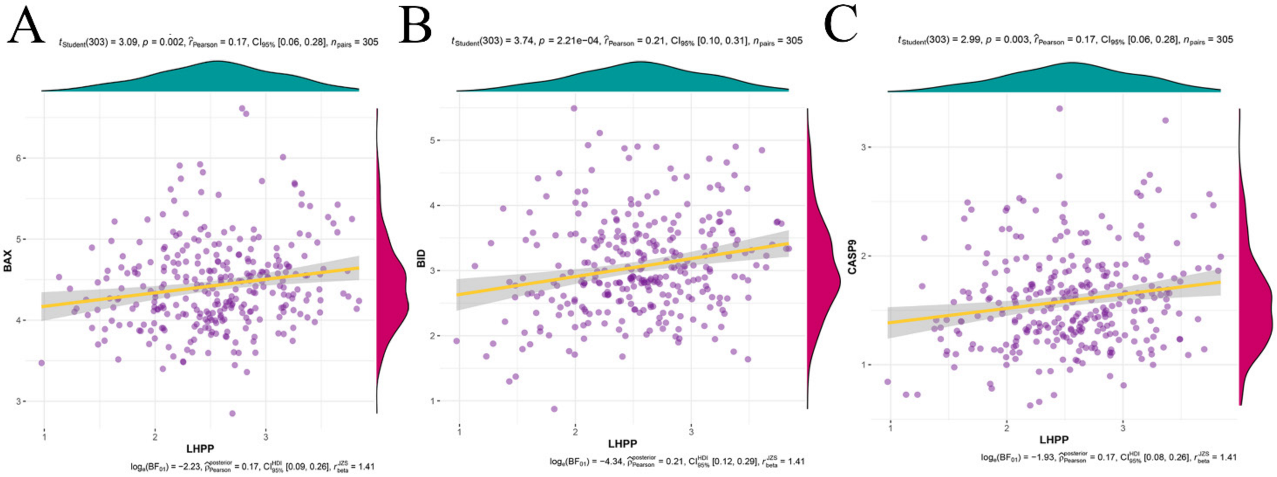

Supplement 3. (A, B, C) The correlation between the expression of LHPP and apoptotic proteins in the TCGA dataset.
